# Shifts in total medical expenses by health coverage changes among the low-income, medically vulnerable population in South Korea

**DOI:** 10.1265/ehpm.24-00409

**Published:** 2025-05-10

**Authors:** Ilsu Park, Kyounga Lee

**Affiliations:** 1 Department of Healthcare Management, Dong-eui University, 176 Eomgwang-ro, Busanjin-gu, Busan 47340, Republic of Korea; 2 College of Nursing, Gachon University, 191 Hambangmoe-ro, Yeonsu-gu, Incheon 21936, Republic of Korea

**Keywords:** Medical aid, Medical expenses, Medically vulnerable population, Health coverage, Healthcare utilization

## Abstract

**Background:**

Medical Aid (MA) beneficiaries, belonging to low-income and vulnerable groups, tend to utilize more healthcare services than patients covered by general health insurance. This study aimed to investigate shifts in medical expenses among South Korean MA beneficiaries from 2010 to 2020 in response to changes in health coverage.

**Methods:**

This study was a retrospective cohort study that involved analyzing data from 354,289 MA beneficiaries aged 20 years and older as of 2010 whose healthcare utilization data could be tracked up to 2020. The impact of changes in health coverage of MA beneficiaries on the increase in medical expenses was analyzed with multiple logistic regression analysis.

**Results:**

The findings revealed that the group maintaining their MA eligibility had a higher rate of increase in medical expenses compared to those transitioning from MA to National Health Insurance (NHI). Even after adjusting for covariates, the likelihood of an increase in total annual medical expenses was more than 1.4 times higher for the MA maintenance group. However, the group that maintained MA also had higher initial healthcare expenses, indicating poorer health status, compared to the group that transitioned to NHI.

**Conclusion:**

In the public healthcare domain, such as MA, it is crucial to enhance access to necessary healthcare services while preventing unnecessary medical treatments. There is a need for systemic improvements to ensure that low-income, medically vulnerable groups can appropriately use the healthcare services they require to achieve high-value health outcomes.

## 1. Background

Many countries have implemented programs to ensure individuals with low-income and medical needs can access healthcare services at minimal or no cost. In the United States, Medicaid provides a range of medical services to the impoverished population, while Medicare offers universal health coverage to individuals aged 65 and older [1]. Similarly, in South Korea, the National Health Insurance (NHI) system mandates all citizens to contribute a monthly health insurance premium proportional to their income to the NHI Service Cooperation, which is operated by the government. When they require healthcare services, the citizens are responsible for paying only about 10%–20% of the total medical costs as out-of-pocket (OOP) expenses [2]. However, for the low-income population that is unable to afford monthly health insurance premiums, the Medical Aid (MA) program allows them to utilize medical services at nearly no cost when necessary. MA beneficiaries are categorized into Type I and Type II; Type I includes socially vulnerable or low-income individuals without working capacity, while Type II encompasses low-income individuals with earned income [3]. Due to their inability to afford medical expenses, MA Type I beneficiaries are only required to pay an OOP cost of less than 1 USD for outpatient visits, with no OOP cost for hospitalizations [3].

There are several reasons why individuals with low-income tend to utilize healthcare services more frequently [4]. Economic hardships can result in inadequate access to proper nutrition, housing, and healthcare, all of which can negatively impact health. Consequently, this can escalate the need for healthcare services. Moreover, there exists a close relationship between persistent poverty and health, wherein poor health status can result in increased healthcare utilization and further impoverishment due to lower economic activity [5]. These factors can make it challenging for people who are consistently in the low-income bracket to maintain their health or prevent diseases. Therefore, the government must provide better access to healthcare services, allocate more resources for the impoverished population, and offer support for better health outcomes [6].

However, decreased OOP cost leads to unnecessary healthcare utilization and overutilization. In the United States, excessive healthcare utilization by Medicaid and Medicare patients has been a persistent issue [7]. With the expansion of Medicaid coverage, there has been a high rate of emergency room overutilization among Medicaid beneficiaries, resulting in increased healthcare expenses [8]. Additionally, it is estimated that 14%–25% of Medicare beneficiaries and 8% of commercially insured adults have experienced at least one instance of healthcare service overutilization per year [9]. Reports indicate that Medicare and Medicaid account for 21% and 18% of total national health expenditure, respectively [10]. Overutilization of healthcare services has been identified as the main cause of high costs in the healthcare system, leading to negative outcomes for patients, such as physical, psychological, social, and financial burdens, and dissatisfaction with healthcare [9, 11].

In South Korea, the continuous increase in medical expenses for MA beneficiaries has posed a financial strain on the government. MA beneficiaries constitute about 2.9% of the total population but account for 10.5% of the total annual healthcare expenses [2]. Compared to the general population covered by the NHI, the annual per capita medical expenses of MA beneficiaries are about three times higher [3].

Policymakers are well aware of the high healthcare utilization by MA beneficiaries. Overutilization of healthcare services is influenced by the medical and patient cultures, the practice environment and incentives, as well as personal experiences for both clinicians and patients [12]. In particular, when patients face low OOP costs and have unrestricted access to healthcare services, both clinicians and patients are prone to demanding more healthcare services [13]. Some countries, instead of reducing OOP costs for low-income patients, restrictions have been imposed on excessive utilization of healthcare services to prioritize high-value healthcare [14]. However, in South Korea, healthcare services are provided based on a Fee-for-Service (FFS) system without institutional restrictions on healthcare service utilization. There is no difference in the allowed healthcare service utilization between MA beneficiaries and other patients. Hence, MA beneficiaries can use more healthcare services without significant constraints.

The question of whether MA beneficiaries utilize more healthcare services due to their circumstances or exploit systemic loopholes to overutilize services remains unanswered. However, the issue of increased medical expenses for MA beneficiaries, attributed to the absence of mechanisms for controlling and managing healthcare service utilization, has been consistently raised [2, 3, 15]. This is due to the characteristics of low-income groups, including limited access to primary care, low interest in preventive measures, and the ability to use medical services excessively without significant financial burden when health conditions deteriorate [2, 8]. In this study, we aim to investigate the association between health coverage and the increase in medical expense among MA Type I beneficiaries, representing the low-income, medically vulnerable population in South Korea, by conducting a longitudinal analysis spanning 11 years.

## 2. Methods

### 2.1. Study design

This study is a retrospective cohort study analyzing the effects of changes in health coverage on increased medical expenses in the low-income population.

### 2.2. Study sample

This study utilized data from 1,530,301 MA beneficiaries as of 2010. Of these, 1,245,448 individuals were eligible for follow-up until 2020. To align with the study objectives, we excluded the following individuals: (1) those younger than 20 years old, (2) those who became MA beneficiaries for reasons other than low income, (3) MA Type II beneficiaries classified as low income but capable of working, and (4) those who experienced more than two transitions between MA and the NHI system.

**Fig. 1:**
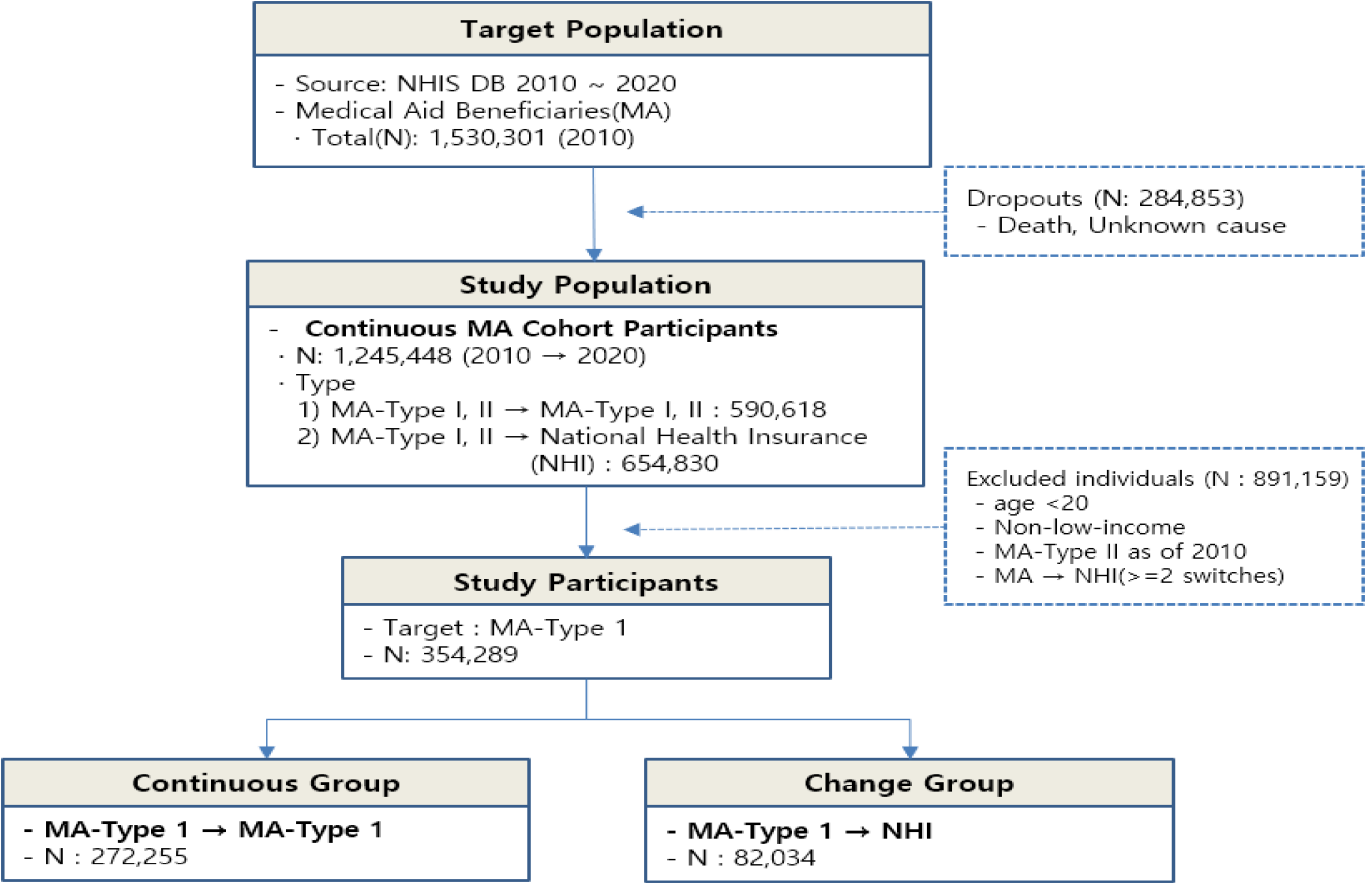
Flowchart of the study sample selection process

After applying these exclusion criteria, the final study sample comprised 354,289 individuals. Among them, 272,255 individuals consistently retained their MA Type I status from 2010 to 2020, whereas 82,034 individuals transitioned from MA Type I to NHI during the study period (Fig. 1). This final sample represented 23.2% of all MA beneficiaries in South Korea in 2010.

### 2.3. Data collection and ethical considerations

Since this study used customized anonymized research data obtained from the Korea National Health Insurance Service (NHIS), it received a review exemption from the Institutional Review Board of D University (IRB No. DIRB-202206-HR-W-10). The NHIS provided de-identified national healthcare utilization data for policy and academic research purposes.

### 2.4. Study measures

Health coverage of the participants was classified into two groups: individuals who continuously maintained their MA Type I eligibility from 2010 to 2020 (continuous group) and individuals who were MA Type I beneficiaries in 2010 but whose health coverage changed to NHI during the follow-up period (change group).

General characteristics included gender, age, and residential area. Additionally, the presence of diseases that could contribute to increased medical expenses was investigated, including rare diseases, mental and behavioral disorders (including dementia), renal diseases, cancer, and chronic diseases (hypertension, diabetes, hyperlipidemia). Diseases were classified according to the Korean Standard Classification of Diseases, which reflects the latest changes in the International Classification of Diseases (ICD-10) and the International Classification of Diseases for Oncology (ICD-O-3). Participants were classified as having a disease if they received treatment for that disease during the follow-up period. Furthermore, participants were considered to have a disability if they were diagnosed with a disability during the follow-up period.

Health screenings are activities that prevent a sudden increase in healthcare service utilization. Therefore, we also surveyed whether the participants underwent a health screening during the follow-up period. To determine whether the participants had a caregiver, we investigated whether they lived alone. Lastly, the income quintiles of the participants in 2010 and 2020 were analyzed, and participants were categorized as having increased income if their income quintile rose, or as having maintained/decreased income if their income quintile remained the same or decreased.

Medical expenses were defined as the total annual medical expenses for each participant. The value was divided into 10 deciles; participants were classified as having increased expenses if their expense decile rose in 2020 compared to that in 2010, and as having maintained/decreased expenses if their expense decile remained the same or decreased in 2020 compared to that in 2010. During the follow-up period, the decile distribution of total annual medical expenses showed a nearly monotonic upward trend. This pattern suggests a consistent increase in medical expenses over time. The difference in deciles between 2010 (baseline) and 2020 (endpoint) serves as a robust indicator of long-term changes in medical expenses. By focusing on this measure, the study minimizes short-term fluctuations and potential noise from intermediate years, allowing for a more accurate assessment of sustained growth in medical expenses.

### 2.5. Statistical analysis

The participants’ health coverage, general characteristics, presence of diseases, disabilities, health screenings, living arrangements (living alone), and income increase were presented using means, standard deviations, frequencies, and percentages. The association between these factors and changes in medical expenses was analyzed using χ^2^-tests and t-tests. The impact of changes in health coverage of MA beneficiaries on the increase in medical expenses was analyzed using three models with multiple logistic regression analysis. Model 1 was adjusted for diseases and disabilities that could directly affect participants’ medical expenses, while Model 2 was further adjusted for health screenings, living arrangements, and income increases, which could prevent an increase in medical expenses. Finally, Model 3 included all variables, including general characteristics (gender, age, residential area), to examine the impact of changes in participants’ health coverage on the increase in medical expenses. All data analyses were performed using SAS software (version 9.4; Cary, NC, USA).

## 3. Results

### 3.1. Increase in medical expenses according to participant characteristics

Among the 354,289 beneficiaries of MA Type I tracked over 11 years from 2010, 36.4% (129,037 participants) experienced an increase in their medical expense decile. In terms of changes in health coverage, 272,255 participants (76.8%) continuously maintained their MA eligibility (continuous group), while 82,034 participants (23.2%) transitioned from MA to NHI (change group). About 38.4% in the continuous group and 29.7% in the change group had an increase in their medical expense decile (χ^2^ = 2,066.941, *p* < 0.001). In terms of participant characteristics, a greater proportion of female participants experienced an increase in medical expenses, with over 40% of those aged 60–70 years experiencing an increase in their medical expense decile. In terms of health conditions, 40.4% of participants diagnosed with cancer experienced an increase in medical expenses (Table 1).

**Table 1:** Change in medical expenses according to participant characteristics

**Variable**			**Total** **(n = 354,289)**		**Change in medical expenses**				

					**Increased** **(n = 129,037)**		**Same/Decreased** **(n = 225,252)**		**χ^2^/t** **(*p*-value)**
		
			**n**	**%**	**n**	**%**	**n**	**%**	
Health coverage		MA (continuous group)	272,255	76.8	104,652	38.4	167,603	61.6	2,066.941 (<0.001)
		NHI (change group)	82,034	23.2	24,385	29.7	57,649	70.3	

Gender		Male	136,351	38.5	47,215	34.6	89,136	65.4	308.028 (<0.001)
		Female	217,938	61.5	81,822	37.5	136,116	62.5	

Age as of 2010 (yr)		Mean ± SD	58.2 ± 14.9		60.4 ± 14.4		57.0 ± 15.5		65.798 (<0.001)

		20–29	13,928	3.9	3,643	26.2	10,285	73.8	4,854.233 (<0.001)
		30–39	28,645	8.1	8,127	28.4	20,518	71.6	
		40–49	65,583	18.5	19,901	30.3	45,682	69.7	
		50–59	68,462	19.3	23,400	34.2	45,062	65.8	
		60–69	76,163	21.5	31,889	41.9	44,274	58.1	
		70–79	84,070	23.7	35,530	42.3	48,540	57.7	
		≥80	17,438	4.9	6,547	37.5	10,891	62.5	

Residential area		Metropolitan	151,355	42.7	56,609	37.4	94,746	62.6	109.611 (<0.001)
		Nonmetropolitan	202,934	57.3	72,428	35.7	130,506	64.3	

Disease	Rare diseases	Yes	40,288	11.4	15,148	37.6	25,140	62.4	27.236 (<0.001)
		No	314,001	88.6	113,889	36.3	200,112	63.7	

	Mental and behavioral disorders	Yes	222,172	62.7	83,358	37.5	138,814	62.5	310.292 (<0.001)
		No	132,117	37.3	45,679	34.6	86,438	65.4	

	Renal diseases	Yes	24,892	7.0	9,518	38.2	15,374	61.8	38.121 (<0.001)
		No	329,397	93.0	119,519	36.3	209,878	63.7	

	Cancers	Yes	36,733	10.4	14,854	40.4	21,879	59.6	285.490 (<0.001)
		No	317,556	89.6	114,183	36.0	203,373	64.0	

	Chronic diseases	Yes	248,567	70.2	98,749	39.7	149,818	60.3	3,931.453 (<0.001)
		No	105,722	29.8	30,288	28.6	75,434	71.4	

Disability		Yes	218,607	61.7	83,760	38.3	134,847	61.7	884.225 (<0.001)
		No	135,682	38.3	45,277	33.4	90,405	66.6	

Health screenings		Yes	199,883	56.4	73,607	36.8	126,276	63.2	32.270 (<0.001)
		No	154,406	43.6	55,430	35.9	98,976	64.1	

Living alone		Yes	279,718	79.0	102,136	36.5	177,582	63.5	4.913 (0.027)
		No	74,571	21.0	26,901	36.1	47,670	63.9	

Income		Increase	33,690	9.5	9,365	27.8	24,325	72.2	1,195.720 (<0.001)
		Same/Decreased	320,599	90.5	119,672	37.3	200,927	62.7	

### 3.2. Changes in total medical expenses by health coverage change

**Fig. 2:**
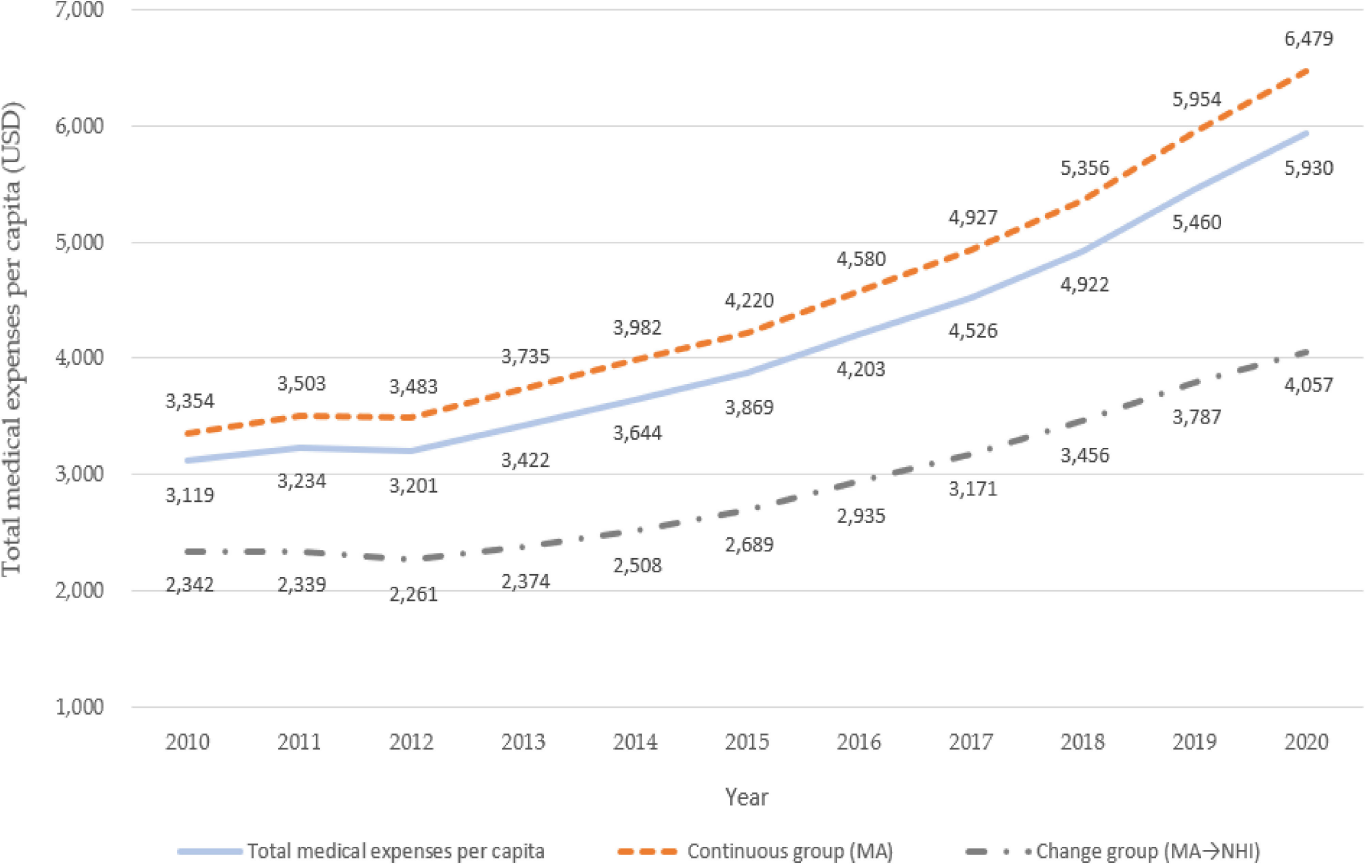
Changes in total medical expenses by health coverage change

The total annual per capita medical expenses increased 1.93-fold from USD 3,354 in 2010 to USD 6,479 in 2020 in the continuous group, while it rose 1.73-fold from USD 2,342 to USD 4,057 in the change group (Fig. 2).

### 3.3. Effects of changes in health coverage on increased medical expenses among MA beneficiaries

Table 2 shows the effects of MA eligibility on increased medical expenses. Model 1 examined the odds of increased medical expenses based on MA eligibility status, after controlling for participants’ underlying diseases. Model 2 additionally controlled for health screenings, living alone, and increased income. Model 3 additionally controlled for participants’ general characteristics (gender, age, region). In all models, individuals who maintained MA eligibility were 1.437–1.466 times more likely to experience increased medical expenses compared to those whose health coverage transitioned to NHI (*p* < 0.001).

**Table 2:** Effects of changes in health coverage on increased medical expenses

**Variable**	**Level**	**Model 1^a^** **(Health model)**			**Model 2^b^** **(Prevention model)**			**Model 3^c^** **(Total model)**		
		
		**OR**	**95% CI**	***p*-value**	**OR**	**95% CI**	***p*-value**	**OR**	**95% CI**	***p*-value**
Medical Aid eligibility	Changed	1.000			1.000			1.000		
	Maintained	1.466	1.441–1.492	<.001	1.437	1.410–1.465	<.001	1.455	1.418–1.473	<.001

Fit statistics	AIC	457,917.111			457,561.208			456,135.029		
	C-statistics	0.580			0.581			0.590		

AIC: Akaike Information Criterion.

^a^Adjustment variable of Model 1: Disease (rare diseases, mental and behavioral disorders, renal diseases, cancers, chronic diseases) and disability.

^b^Adjustment variable of Model 2: Disease (rare diseases, mental and behavioral disorders, renal diseases, cancers, chronic diseases), disability, health screenings, living alone, and income.

^c^Adjustment variable of Model 3: Disease (rare diseases, mental and behavioral disorders, renal diseases, cancers, chronic diseases), disability, health screenings, living alone, income, and demographic characteristics (gender, age, and residential area).

## 4. Discussion

This study retrospectively compared the annual medical expenses between low-income and medically vulnerable individuals designated as MA Type I beneficiaries in 2010, who maintained their MA eligibility until 2020(continuous group), and those whose health coverage changed to NHI(change group).

From 2010 to 2020, the total annual per capita medical expenses increased by 1.93 times for the group that maintained MA eligibility and 1.73 times for the group that transitioned to NHI. Among all participants, 36.4% (129,037 out of 354,289) experienced an increase in their medical expense decile. Specifically, 38.4% of the continuous MA group and 29.7% of the NHI change group saw an increase in medical expense decile. This suggests that those who continuously retained their MA eligibility utilized more healthcare services compared to the change group. Moreover, logistic regression analysis revealed that the continuous MA group had 1.4 times higher odds for an increase in their medical expense decile compared to the health coverage change group, even after adjusting for variables related to diseases, health screenings, living alone, income, and general characteristics. This suggests that the group consistently maintaining their MA eligibility utilized more healthcare services, even when accounting for the impact of these potential confounders on healthcare utilization. These findings align with a previous study that examined the increase in medical expenses of MA beneficiaries over two years [2].

Government policies can be attributed as one reason for the increase in medical expenses. Since the inception of Medicare and Medicaid, healthcare providers felt comfortable increasing their prices, knowing that the government, rather than individuals, bears the expense. From the patient’s perspective, the reduced financial burden for medical expenses tends to encourage the utilization of more healthcare services. The fact that individuals enrolled in high-deductible health plans (HDHP) exhibited lower healthcare utilization, due to higher OOP costs, supports our findings [13]. However, while HDHPs can reduce healthcare utilization, they can also result in the postponement or avoidance of necessary healthcare services due to financial concerns, potentially leading to more severe health challenges later on. The rise in medical expenses underscores the importance of distinguishing between high-value and low-value healthcare services [16]. Although spending on necessary healthcare services is valuable, unnecessary medical expenses should be curtailed. The World Health Organization defines integrated health as “the organization and management of health services so that people receive the care they require, promptly and conveniently, with outcomes aligned to their needs, and in a cost-effective manner [17].” This implies that integrated health involves effective communication about patient care among healthcare providers, management, and support teams. In a disjointed healthcare system, the lack of coordination can result in patients undergoing redundant tests and utilizing more healthcare services than necessary, yet experiencing poorer health outcomes [18, 19]. For beneficiaries of MA in South Korea, inadequate management and coordination between healthcare service providers and beneficiaries may result in excessive utilization of low-value healthcare services. This, in turn, contributes to the increase in medical expenses, highlighting the need for serious consideration to identify points for systemic improvements. Research on the causes of unnecessary utilization indicates that not only the coverage of the patient’s insurance but also the reimbursement mechanism, can have an impact [20]. South Korea uses an FFS system, where healthcare providers generate more revenue by providing more services. This can significantly lead to an abundance of redundant tests, overtreatment, or overprescription, especially among patients with a low potential for improved health outcomes. A more common strategy in the public sector, such as MA, is to consider shifting from volume-based payments to value-based payments [16]. If excessive healthcare utilization arises due to low OOP costs, it is important to control the loopholes in the system to ensure that services are provided only where and to the extent needed.

The second point to note is that the total annual per capita medical expenses were 1.43 times higher in the continuous group (USD 3,354) compared to the change group (USD 2,342), even in 2010 when all participants were MA beneficiaries. In other words, despite both being MA beneficiaries in the first year of follow-up, the group that continued to maintain MA eligibility was already incurring more medical expenses. This suggests that they were in a less healthy condition, requiring them to utilize more healthcare services despite having the same level of access to healthcare. This also implies that the group with higher medical expenses and poorer health status continuously remained in a lower economic status. The positive correlation between income and health has been well-documented [21, 22]. While it is not clear which of the two is the primary factor, it is evident that they influence each other. According to the Relative Income Hypothesis, the utilization levels of key resources and medical services vary based on income class, resulting in health disparities stemming from discrepancies in the capacity to possess and access these resources [21]. Conversely, studies analyzing the impact of health on income have indicated that health exerts a stronger influence on income within the extremely poor population [22]. In South Korea, the MA program provides healthcare services for the lowest-income group. The relationship between income and health forms an inverted U-shape, showing a positive correlation between income and health until a certain threshold is reached [23]. Good health enhances employment opportunities, resulting in increased income. Conversely, poor health tends to confine individuals to the low-income bracket. Individuals with frail health may struggle to generate sufficient income, rendering them eligible for MA. Consequently, they may face an elevated risk of requiring more frequent treatments and encountering emergencies [8]. Hence, the increased utilization of healthcare services by the continuous MA group may not solely stem from system loopholes but also their heightened need for healthcare services. This study discovered that the number of participants who continuously maintained their MA eligibility is approximately 3.32 times higher than those whose health coverage changed to NHI. In other words, the rate of a complete shift in health coverage from MA Type I to NHI over ten years is relatively low. This suggests that healthcare policies should focus on providing systematic support to facilitate this transition from an MA-eligible state to an NHI-eligible state (i.e., equipping them with the capacity to earn income). This can be achieved by reducing inequalities in acquiring and utilizing health-related resources, facilitating appropriate treatments to boost health outcomes, providing systematic support to improve the health outcomes of beneficiaries, and enabling MA beneficiaries to qualify for NHI (i.e., having the capacity to generate income).

Finally, attention should be directed toward the differences in the rate of increase in medical expenses associated with participants’ diseases and geographical regions. Among the MA beneficiaries in this study, 62.7% were diagnosed with mental and behavioral disorders (including dementia) during the 11-year follow-up period. In the US, about half of all Americans are reported to have been diagnosed with a mental illness or disorder at least once in their lifetime [24]. However, in South Korea, the lifetime prevalence of mental disorders (excluding dementia) is 27.8% [25], and the prevalence of dementia among the older adult population aged 65 years and above is about 7.5% [26]. Therefore, the prevalence of mental and behavioral disorders identified in this study is nearly twofold higher. Low-income individuals experience a higher rate of mental illness, emphasizing the importance of mental health treatment [24, 27]. The medical expenses associated with mental illness continue to increase [25, 26]. Without timely treatment, more expensive interventions, such as hospitalization, may be necessary. Therefore, it is crucial to focus on the management of mental disorders among MA beneficiaries [24]. Considering regional differences, the rate of increase in medical expenses was higher among residents in metropolitan areas than in nonmetropolitan areas. This indicates that healthcare utilization is higher in cities where resources are concentrated. A previous study has also revealed that medical abuse is more common in urban areas [9]. Healthcare utilization includes supplier-induced demand, and high availability and accessibility of healthcare can lead to healthcare abuse [20]. One of the key components of managing unnecessary healthcare services is identifying and addressing the root causes of overutilization. As the MA program is funded with taxpayers’ money, it is crucial to reinforce strict management measures to prevent unnecessary waste.

This study has several limitations. First, participants who experienced multiple fluctuations between MA Type I and NHI were excluded, introducing the potential for selection bias. By omitting these individuals, the study may not fully capture the complexities of healthcare utilization among those with unstable coverage, which could lead to an overestimation or underestimation of the relationship between health coverage changes and medical expenses. Second, the study did not account for the timing of transitions from MA Type I to NHI during the follow-up period. This omission introduces temporal ambiguity, as differences in healthcare utilization may exist between individuals who changed their coverage early in the study period and those who transitioned later. Third, the possibility of reverse causation cannot be ruled out. That is, individuals’ poor health status and higher medical expenses may have influenced their health coverage status rather than the other way around. Fourth, healthcare utilization was measured solely based on total annual medical expenses. Since outpatient, inpatient, and emergency department visits have distinct implications for healthcare utilization, future research should incorporate more detailed measures to capture utilization patterns across different healthcare settings. Finally, various factors that may influence healthcare utilization but were not included in this study should be considered in future research.

Despite these limitations, this study presents a robust analysis of the impact of health coverage changes on rising medical expenses by leveraging a large-scale dataset, an extended follow-up period, high-quality administrative records, and rigorous adjustment for confounding variables. The findings provide valuable insights that can inform future enhancements in healthcare policy and resource allocation.

## 5. Conclusion

This study aimed to analyze the impact of changes in health coverage on the variation in healthcare expenses among MA beneficiaries. The findings revealed that participants who consistently maintained MA eligibility not only utilized more healthcare services initially but also exhibited a higher rate of increase in healthcare expenses compared to those whose health coverage changed to NHI. After adjusting for covariates, the probability of an increase in healthcare expenses was more than 1.4 times higher for individuals retaining their MA eligibility. This suggests that maintaining MA eligibility is associated with increased healthcare utilization. While the MA program is a crucial system designed to ensure universal health coverage for the low-income population and prevent health disparities, it is imperative to establish mechanisms for management and control to prevent its excessive utilization. Moreover, given the association between poor health status and a greater probability of remaining in a low-income group, systematic management, and education are essential for improving the health status of MA beneficiaries. Systematic excessive healthcare utilization has been documented [9], underscoring the need for policymakers and healthcare system leaders to address the underlying factors driving overutilization. This study’s findings should catalyze fostering high-value health outcomes.

## Declarations

### Availability of data and materials

No data are available. This study used customized anonymized research data that cannot be shared without ethical approval. The Korea National Health Insurance Service (NHIS), the data provider, precludes sharing of aggregated data sets. Analysis data sets are stored according to the ethical approval and access can only be provided to researchers who have received approval from the ethics committee.
